# A trial of hypoimmune allogeneic CD19-targeting T cells in refractory systemic lupus erythematosus

**DOI:** 10.1093/ckj/sfag068

**Published:** 2026-03-10

**Authors:** Vanja Ivković, Augusto Vaglio

**Affiliations:** Department of Health, Medicine and Caring Sciences, Linköping University, Linköping, Sweden; Department of Nephrology, Hypertension, Dialysis and Transplantation, University Hospital Center Zagreb, Zagreb, Croatia; Faculty of Health Studies, University of Rijeka, Rijeka, Croatia; Department of Biomedical, Experimental and Clinical Sciences “Mario Serio”, University of Florence, Florence, Italy; Nephrology and Dialysis Unit, Meyer Children’s Hospital IRCCS, Florence, Italy

Chimeric antigen receptor (CAR)-T cell therapy targeting B cells has demonstrated efficacy in severe autoimmune diseases, including systemic lupus erythematosus (SLE) [1, 2]. There are, however, important challenges associated with CAR-T cell therapy, including its high cost, lengthy and complex manufacturing process, and the necessity for patients to temporarily discontinue immunosuppressive therapy, which might pose major risks [3]. Findings from a previous study demonstrated that a T-cell receptor (TCR) complex–based chimeric antigen receptor, termed synthetic TCR and antigen receptor (STAR), improves antigen sensitivity, proliferation and persistence compared with classical CAR-T cells, and has lower dysfunction and toxicity [4]. A recently published first-in-human Phase I trial aimed to explore the safety, efficacy and mechanisms of STAR-T cells application in patients with severe refractory SLE and lupus nephritis (LN) [5]. The trial included five patients with refractory SLE and LN. The primary endpoints were safety and SLE responder index 4 (SRI-4) at Month 3. Secondary endpoints included SRI-4 at Month 6, other clinical remission measures and several patient-reported outcomes.

Briefly, YTS109, a CD19-targeted STAR-T cell product, was generated from the peripheral blood mononuclear cells of a healthy donor through a multistep process, most importantly a series of genetic modifications performed using CRISPR-Cas9 to knock out genes responsible for graft-versus-host disease (GVHD), immune recognition and T-cell persistence, namely *TCRαβ, PD1, HLA-A, HLA-B* and *CIITA*, and inserting the STAR construct into TRAC (T-cell receptor alpha constant), a gene region for the TCR (Fig. 1A). All immunosuppressive therapy was discontinued before administering YTS109, with tapering to a minimal physiologically dependent dose being performed in patients on high-dose glucocorticoids. After lymphodepletion with fludarabine and cyclophosphamide, the patients received a single YTS109 infusion on Day 0.

**Figure 1:**
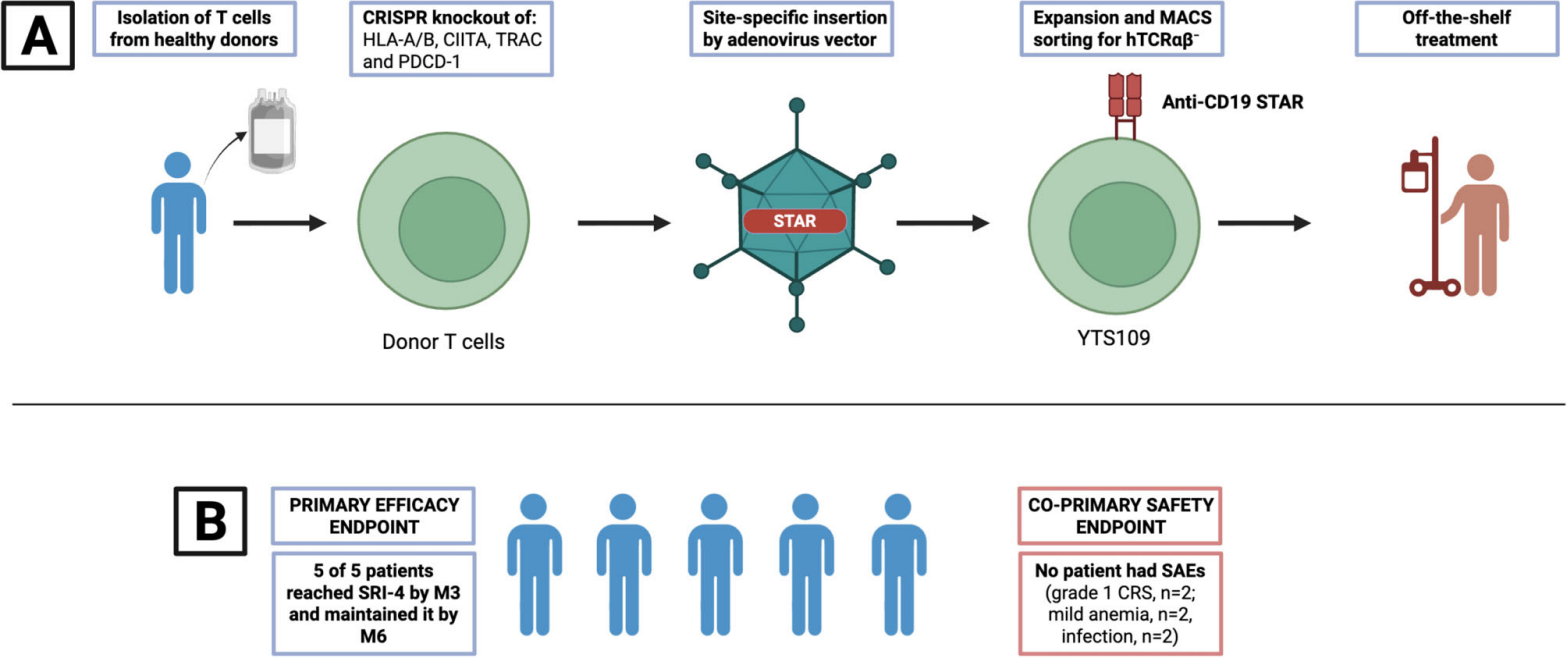
(**A**) Key steps in the production of YTS109: Step 1, T cells are isolated from a healthy donor; Step 2, knockout of genes encoding HLA A/B, CIITA, TRAC and PD-1; Step 3, site-specific knock in of anti-CD19 STAR into the TRAC locus using adenovirus vector; Step 4, *in vitro* cell expansion and magnetic-activated cell sorting for TCR^−^ cells; and Step 5, treatment is given to patients. (**B**) All patients reached SRI-4 by Month 3 (primary efficacy endpoint) and maintained it by Month 6, and no patient had severe adverse events (co-primary safety endpoint). CD, cluster of differentiation; CIITA, class II; major histocompatibility complex; transactivator; CRS, cytokine release syndrome; HLA, human leukocyte antigen; M, month; MACS, magnetic-activated cell sorting; PD-1, programmed cell death protein 1; SAE, severe adverse event; SR4, SLE responder index.

All patients achieved SRI-4 at Month 3 (primary efficacy endpoint) with a maintained response up to Month 6. Disease activity decreased in all patients, with four reaching a Safety of Estrogen in Lupus National Assessment–Systemic Lupus Erythematosus Disease Activity Index (SELENA-SLEDAI) score of zero. One patient experienced a mild flare-up. There were no serious adverse events (co-primary safety endpoint), including signs of GVHD (Fig. 1B).

Aside from these, there were several findings regarding secondary and exploratory endpoints, most importantly: (i) B-cell loss was rapid and persisted for 1–2 months with all patients reconstituting B cells afterwards; (ii) some patients achieved clearance of anti-dsDNA maintaining near-zero levels throughout the study and normalization of C3 and C4 serum levels; (iii) most patients had substantial 24-h proteinuria levels at baseline, with two patients achieving complete renal remission (<500 mg/day) at follow-up. Importantly, two patients who achieved clinical remission free of continued immunosuppressant use and underwent follow-up kidney biopsies showed histologic evidence of renal recovery.

Moreover, the authors evaluated compositional changes in immune cells and clonal dynamics of T and B cells, and found a rapid and deep clearance of B cells followed by a reconstitution of a much larger B-cell population after 2 months. The reconstituted B cells consisted mostly of immature and naïve B cells with low level of antigen-experienced cells which shifted to mostly naïve B cells as time progressed. As compared with CAR-T cells, hypoimmunogenic allogeneic YTS109 appeared to have a comparable ability to induce B-cell depletion but a shorter persistence in the peripheral blood (as they disappeared after 1–2 months); this is a theoretical advantage as it potentially reduces the risk of infections and malignant transformation of T cells.

While the study has several limitations, including lack of a comparator arm, small sample size and short follow-up, it provides evidence for the safety and potential efficacy of this therapy in highly selected, refractory SLE patients. Particularly interesting is the fact that the hypoimmune allogeneic cells disappeared quite early while being able to achieve a profound B-cell depletion, and that B cells reconstituted 2–3 months after achieving immune reset which helps avoid unnecessary long-term immune suppression and severe infections. Overall, the trial expands on our knowledge of cell therapies in SLE and establishes a rationale for further exploration of the use of YTS109 in autoimmune diseases.

## References

[1] Mougiakakos D, Krönke G, Völkl S et al. CD19-targeted CAR T cells in refractory systemic lupus erythematosus. N Engl J Med. 2021;385:567–9. 10.1056/NEJMc2107725 34347960

[2] Müller F, Taubmann J, Bucci L et al. CD19 CAR T-cell therapy in autoimmune disease—a case series with follow-up. N Engl J Med. 2024;390:687–700. 10.1056/NEJMoa2308917 38381673

[3] Schett G, Müller F, Taubmann J et al. Advancements and challenges in CAR T cell therapy in autoimmune diseases. Nat Rev Rheumatol. 2024;20:531–44. 10.1038/s41584-024-01139-z 39107407

[4] Liu Y, Liu G, Wang J et al. Chimeric STAR receptors using TCR machinery mediate robust responses against solid tumors. Sci Transl Med. 2021;13:eabb5191. 10.1126/scitranslmed.abb5191 33762437

[5] Wang X, Zhang Y, Wang H et al. Allogeneic CD19-targeting T cells for treatment-refractory systemic lupus erythematosus: a phase 1 trial. Nat Med. 2025;31:3713–24. 10.1038/s41591-025-03899-x 40866583

